# Pharmacokinetic Effects of THC and Melatonin Co‐administration in Elderly Population

**DOI:** 10.1002/alz.095382

**Published:** 2025-01-09

**Authors:** María Paula Naranjo, Fabio Camargo, Jagadeesh S Rao, Laura Delgado‐Murillo, Margarita Venegas, Maria Alejandra Tangarife, María Juanita Arbelaez, Evelyn Gutiérrez, Laura Tatiana Sánchez, Saadia Shahnawaz, Varduhi Ghazaryan, William Julio De Jesus, Claudia Grimaldi, Elliot Hong, Ram Mukunda

**Affiliations:** ^1^ IGC Pharma, Bogota, Bogota Colombia; ^2^ IGC Pharma, Bogotá, Bogotá D.C. Colombia; ^3^ IGC Pharma, Potomac, MD USA; ^4^ IGC Pharma, LLC, Bogota Colombia; ^5^ Santa Cruz Behavioral, Bayamon, PR USA; ^6^ UTHealth Houston McGovern Medical School, Houston, TX USA

## Abstract

**Background:**

Cannabis‐based therapies have gained interest in treating different ailments in the elderly population, including severe or chronic pain, sleep disturbances, and more recently Alzheimer’s Disease. This raises the importance of understanding the influence of age on the pharmacokinetics (“PK”) of delta‐9 tetrahydrocannabinol (“THC”).

IGC‐AD1 comprises THC at a low concentration and melatonin. The PK of THC/Melatonin co‐administration acquires importance since recent therapeutics employ both drugs in treating insomnia or neurodegenerative diseases in the geriatric population, where baseline PK is key to understanding possible changes because of renal or hepatic failures, and accurately adjusting doses to ensure the safety of those drugs.

**Method:**

Data is presented from a Phase 1 Multiple Ascending Dose (“MAD”) study (n = 10 active), on AD patients and compared to PK data for adults. A review was performed including search results from ScienceDirect, CAS, PubMed, SpringerLink, and Google Scholar, using the terms “Pharmacokinetics”, “Absorption”, “transporter*”, “intestinal”, and “elderly”. Inclusion criteria were the indexed nature of the research, and languages (English, Spanish). No temporal inclusion‐exclusion criteria were considered.

**Result:**

A dose of 2.5mg of THC reaches higher maximum plasma concentrations (C_max_ = 2ng/mL), when administered together with 1.5mg of melatonin, and melatonin appears to be metabolized faster when administered with THC. When considering the relationship between the administered dose and C_max (Cmax/dose)_, C_max_ per dose of the co‐administered THC, 0.0008, is significantly higher than other reported PK data for oral cannabis administration 0.0003 (p<0.01).

**Conclusion:**

Melatonin when administered jointly with THC could play a significant role in THC absorption and PK. We hypothesize, pending more studies, that the higher reported dose‐dependent absorption of THC may be related to the effect of melatonin on gastrointestinal transporters and efflux pumps.

